# Blastomycosis-Induced Acute Respiratory Distress Syndrome

**DOI:** 10.7759/cureus.22207

**Published:** 2022-02-14

**Authors:** Maleeha Ajmal, Fahad Aftab Khan Lodhi, Gul Nawaz, Ahmad Basharat, Afifa Aslam

**Affiliations:** 1 Internal Medicine, Marshfield Clinic Health System, Marshfield, USA; 2 Nephrology, University of California, Los Angeles, USA; 3 Internal Medicine, Jinnah Hospital, Lahore, PAK

**Keywords:** acute respiratory distress syndrome (ards), prone ventilation, neurally adjusted ventilatory assist, vv ecmo, pulmonary blastomycosis

## Abstract

Blastomycosis is a systemic mycosis endemic to the Midwestern and South Central United States. Infection is caused by inhaling spores of *Blastomyces dermatitidis* (*B. dermatitidis*) that inhabit soil. Acute respiratory distress syndrome (ARDS) is a rare complication of pulmonary blastomycosis with a significantly high mortality rate. We present a case of blastomycosis associated with severe ARDS treated with traditional prone position ventilation (PPV) and neurally adjusted ventilator assist (NAVA) along with antifungal therapy, steroids, and supportive care in a rural setting with no access to extracorporeal membrane oxygenation (ECMO). This case demonstrates that traditional therapies such as prone position ventilation can help patients with blastomycosis-associated ARDS especially in rural settings where advanced therapies such as ECMO are lacking. The use of NAVA in blastomycosis-associated ARDS needs further research.

## Introduction

Blastomycosis is a fungal infection caused by inhaling spores of a thermally dimorphic fungus *Blastomyces dermatitidis* (*B. dermatitidis*). Pulmonary and cutaneous mycoses are primarily encountered in people living in the Midwestern and South Central United States [1]. In most cases, pulmonary blastomycosis is asymptomatic, causing acute, self-limited disease that often goes unrecognized [2]. Acute respiratory distress syndrome (ARDS) is a rare complication of pulmonary blastomycosis that is seen in 10% of all cases and has reported mortality rates of as high as 89% [3]. More recently, extracorporeal membrane oxygenation (ECMO) has been reported as a successful rescue tool in patients with refractory blastomycosis-associated ARDS [4]. There is a paucity of medical literature regarding the management of blastomycosis-associated ARDS.

This study was presented as poster abstract at the American College of Physicians Wisconsin Chapter Scientific Meeting 2020.

## Case presentation

**Figure 1:**
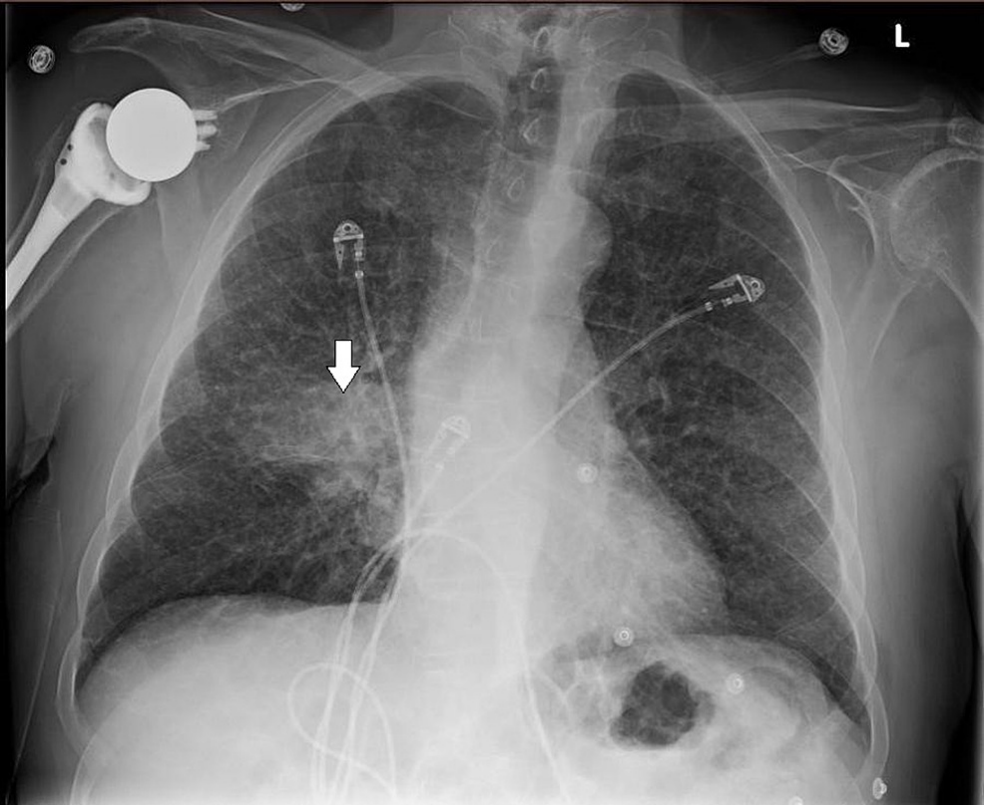
Chest x-ray showing dense right lower lobe infiltrate (arrow)

A 70-year-old male from rural Wisconsin was admitted to the medical intensive care unit due to worsening shortness of breath and acute hypoxic respiratory failure requiring intubation and mechanical ventilation. He was a previous smoker but had no known chronic respiratory issues. His symptoms started several days prior to presentation, with initially dry cough that became productive with worsening shortness of breath. He was feeling generally unwell and had chills but no fevers. He initially presented to a different facility and was found to have hypoxia, elevated white cell count, C-reactive protein, venous lactate, and right lower lobe infiltrate on chest radiograph (Figure 1).

**Figure 2:**
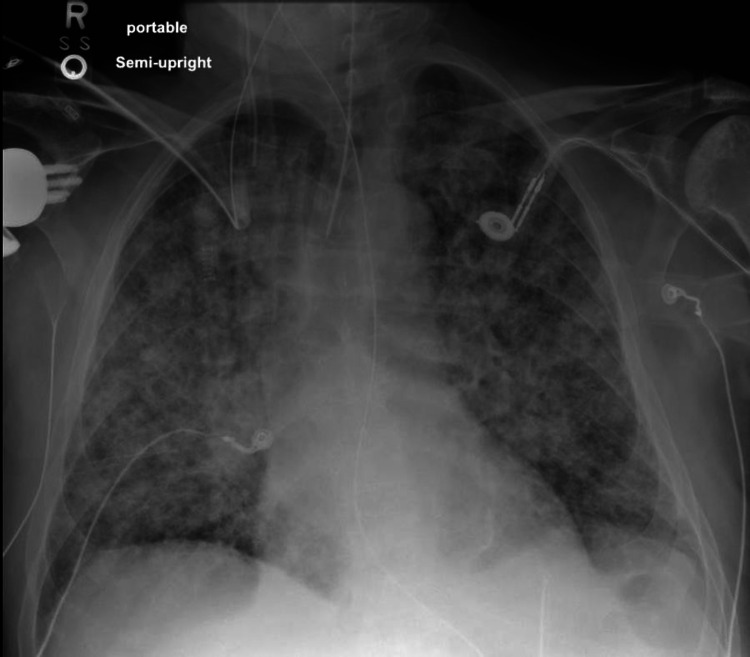
Chest x-ray showing diffuse bilateral pulmonary opacities suggesting the development of ARDS ARDS: acute respiratory distress syndrome

He was diagnosed with community-acquired pneumonia and was started on intravenous ceftriaxone and azithromycin. His condition deteriorated over the next 48 hours with septic shock and worsening respiratory symptoms, development of bilateral lung opacities on chest radiograph, and severe hypoxemia, all suggestive of ARDS, requiring intubation and mechanical ventilation (Figure 2). Later, he was transferred to our tertiary care facility for a higher level of care.

**Figure 3:**
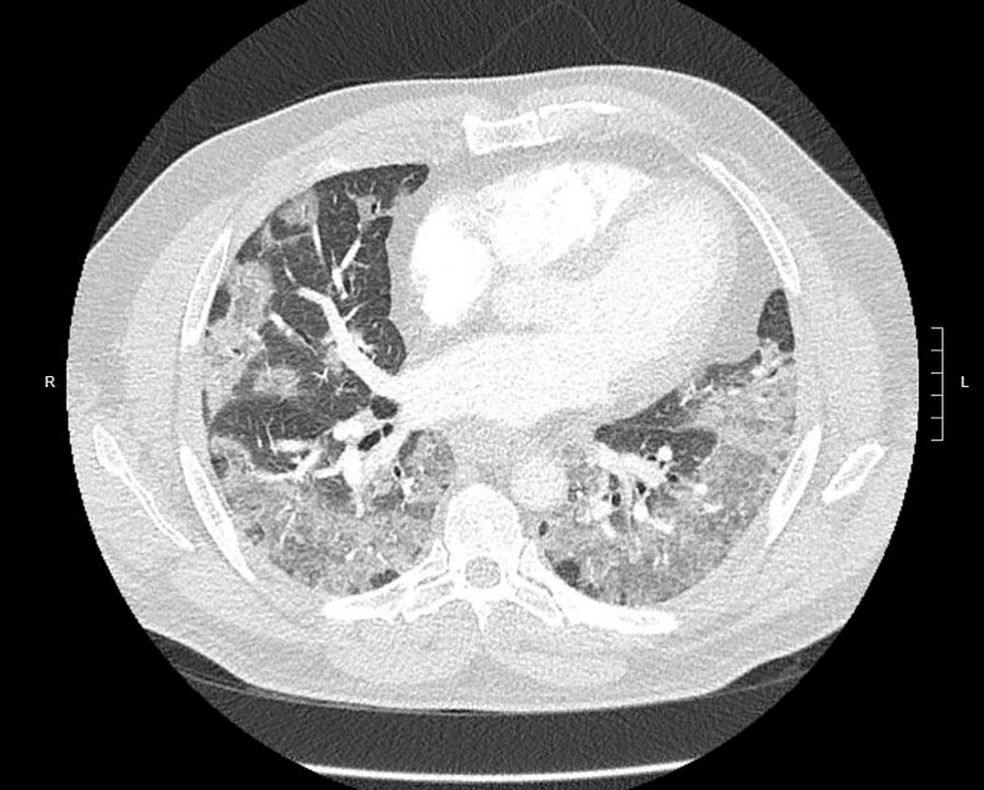
CT chest showing dense bilateral pulmonary infiltrates

Upon presentation to our facility, he was deeply sedated, intubated, had diffuse bilateral lung crackles and skin mottling. He required intravenous norepinephrine and vasopressin infusions to maintain blood pressure with mean arterial pressure (MAP) goal of more than 65 mmHg. He was on broad-spectrum antibiotics with intravenous vancomycin and cefepime. Laboratory investigations showed leukocytosis, acute kidney injury, and severe metabolic acidosis. CT chest showed diffuse bilateral pulmonary infiltrates (Figure 3). Blood cultures showed no growth, a complete respiratory viral panel was negative, and his sputum cultures revealed yeast cells resembling *B. dermatitidis*. He underwent bronchoscopy with bronchoalveolar lavage (BAL), with the specimen again revealing *B. dermatitidis* and ruling out other infectious etiologies.

Antibiotics were changed to liposomal amphotericin B (L-AMB), and he was started on continuous renal replacement therapy to treat severe metabolic acidosis and acute kidney injury. He was continued on assist mode of mechanical ventilation with ARDS protocol including lung-protective, low-tidal volume ventilation. Intravenous (IV) methylprednisolone for severe ARDS (PaO_2_/FiO_2_) was ≤100 mmHg on ventilator settings that included positive end-expiratory pressure (PEEP) ≥5 cm H_2_O. The patient was noted to require a higher inspired oxygen fraction (FiO2), even after deep sedation and muscle paralysis with neuromuscular blockade and fluid restriction policy. A two-dimensional echocardiogram showed normal left ventricular systolic function. Due to lack of access to ECMO in our facility, the decision was made to treat the patient in the prone position. PPV sessions of 16-hours duration per day for a total of three days were begun and stopped after hypoxemia improved. He received aggressive pulmonary toilet with chest vest and nebulized hypertonic saline. However, patient-ventilator dyssynchrony persisted despite multiple modifications to the ventilator mode and settings. He was gradually transitioned to NAVA, which he tolerated well. A tracheostomy was performed, and the patient tolerated a gradual increase in duration and frequency of pressure support via tracheostomy collar before being considered for discharge. He was later discharged to a long-term, acute care facility with continuous positive airway pressure support, the pressure of 5 cm H_2_O with 30% FiO_2_ at night time, and on tracheostomy collar during the day time with 40% FiO_2_. He was treated with L-AMB for 16 days and then transitioned to oral itraconazole for at least six months as recommended by infectious disease specialist. The patient continued to follow-up with his primary care physician and infectious disease specialist upon discharge.

## Discussion

ARDS is a rare and poorly understood complication of pulmonary blastomycosis, and a limited amount of literature is available for healthcare providers to aid in the management of such patients, especially in endemic regions [3]. ARDS complicates up to 10% of cases of blastomycosis, and when it does occur, mortality ranges from 50% to 90%, which is disproportionately high, even when compared to ARDS caused by other entities [4]. To avoid this fatal complication, physicians should have prompt recognition of this disease with the initiation of timely, aggressive treatment, including amphotericin B [1,5].

Rush et al. published the largest retrospective cohort analysis in March 2020, which included 1848 patients with a diagnosis of blastomycosis who presented to the hospital from 2006 to 2014 in the United States; 11.9% of the patients required mechanical ventilation, and they determined mortality of as high as ~40% for patients with blastomycosis requiring mechanical ventilation [6]. Schwartz et al. had also published a large historical case series in 2016 involving 43 critically ill patients with ARDS caused by blastomycosis over a 23-year period who required mechanical ventilation in Manitoba, Canada [7]. In that report, 67% of patients had severe ARDS, with 40% overall mortality rate. A case series by Azar et al. reported 114 cases of blastomycosis, with an ARDS rate of 15% and a mortality rate of 47% among patients who developed ARDS [8]. Ventilatory management has evolved with improvements over time, which include lung-protective, low-tidal volume ventilation [9]. However, the published literature lacks specific guidelines on the treatment of blastomycosis-associated ARDS.

There is sparse literature highlighting the use of adjunctive steroids to suppress the inflammatory response, and no randomized, controlled trials have been performed to support improved outcomes [10,11]. In the case series by Shwartz et al., 50% of the patients received steroids, but there were no reported significantly better outcomes [7]. In the cohort study by Rush et al., there was no description of the percentage of patients receiving steroids due to lack of efficient medication administration recording tools [6].

Early application of PPV was also a key component in our patient’s survival, in our opinion. Prone position ventilation has been studied and identified to be of benefit in severe ARDS, but limited data have been published on its use in blastomycosis-associated ARDS. Prone positioning has been employed since the 1970s to improve hypoxemia in patients with ARDS [12-14]. Passive mechanical ventilation in the supine position caused ventilation distribution primarily to nondependent lung regions where there was low perfusion [15]. As acute respiratory failure is combined with decreased functional residual capacity, and since supine position increases dependent airway closure, Bryan suggested that PPV might engage and stabilize dependent lung segments [16]. The recent publication of the landmark PROSEVA study [17], and consistent results of numerous meta-analyses of randomized controlled trials, describe a clear mortality benefit when prone position strategy is applied early and for prolonged time periods in patients with severe ARDS [18].

NAVA was utilized in our patient. There are currently no published data that show the use of NAVA in ARDS secondary to blastomycosis with a successful outcome. NAVA is a relatively new mode of assistive mechanical ventilation. It utilizes the electrical activity of the diaphragm to trigger and drive inspiratory cycle in proportion to the patient’s effort [19-22]. However, there are studies suggesting that NAVA improves patient-ventilator synchrony and reduces the risk of over- assistance, as well as its use in selective ARDS patients [23,24].

ECMO has also been described as a rescue, supportive measure when patients fail to improve their oxygenation status despite maximum lung-protective ventilatory settings [4]. However, the role of ECMO is yet to be unraveled [25]. Rush et al. sampled 219 patients requiring mechanical ventilation, but none of them received ECMO as salvage therapy [6]. The case series published by Bednarczyk et al. reported four cases of severe ARDS requiring ECMO with 100% survival, suggesting this therapy may be beneficial for salvage therapy, although further studies are needed to confirm these findings [4]. Moreover, there are conflicting data on the use of ECMO in patients with disseminated blastomycosis and septic shock [26].

## Conclusions

The outcome in this patient study is exceptional given the extremely high mortality of blastomycosis-associated ARDS. The patient’s age is another unique factor, given that most previous success stories have been reported in younger populations. Despite a delay in diagnosis, and thus delayed initiation of antifungal therapy, the patient survived with ventilatory support using PPV, NAVA, chest physiotherapy, L-AMB, and IV steroids to suppress the inflammatory response. A great deal of work still needs to be done, and data need to be published to guide management in blastomycosis-associated ARDS regarding the use of traditional methods.
